# The Whole is Greater Than the Sum of Its Parts – A Quality Improvement Project Evaluating the Impact of Peer Study Groups for Preparation Towards MRCPsych Exams

**DOI:** 10.1192/bjo.2026.11348

**Published:** 2026-06-30

**Authors:** Naeem Ali, Leah Riley

**Affiliations:** 1 Midlands Partnership University NHS Foundation Trust, Stafford, United Kingdom; 2 Midlands Partnership University NHS Foundation Trust, Shropshire, United Kingdom

## Abstract

**Aims:**

In 2025, 6366 applicants sat Membership of the Royal College of Psychiatrists (MRCPsych) examinations. High stakes for applicants include cost, difficulty, and career progression. Written exams cost around £500, and roughly £1000 for UK-based CASC (Clinical Assessment of Skills and Competencies). Pass rates range between 35–71.2% for written exams and 44.6–65.3% for UK-based CASC. MRCPsych completion is essential for accessing higher training. Trainees attend school-run MRCPsych programmes to becomerounded clinicians, but some voice concerns around insufficient examination focus and purchase additional study resources.

Post-Covid norms include decentralised and online teaching, which diminished networking opportunities enabling peer study groups forming. Theories from Vygotsky (Zone of Proximal Development Theory), Bandura (Social Learning Theory), and Jean Pol-Martin (Protégé Effect) favour peer-learning principles.

This project evaluated whether locally implemented peer-led study groups blending online and in-person sessions improved perceived examination confidence, preparedness, and syllabus knowledge.

**Methods:**

Plan, Do, Study, Act approach was utilised. A focus group identified group-study themes (preparation, structure, content, feedback) and challenges (format, learning styles, additional needs, resources, geographical spread, availability) and identified aims and outcome measures (Likert scale measuring perceived examination confidence, preparedness, and syllabus knowledge).

Written examination groups started in January 2025. Participants self-arranged weekly online or face-to-face sessions blending peer-led teaching with group discussion answering multiple choice questions from shared question banks. Session frequency increased approaching examinations, and prioritised questions. Face-to-face CASC sessions featuring consultant examiners occurred monthly between March–July 2025. Affordable technology including microphones and video-calling enabled some examiners to assess remotely.

**Results:**

Feedback (39.4% response) on school-led teaching flagged insufficient interactivity,passion, and examination/curriculum focus. School-led CASC session organisation was praised; however, participant anxiety and feedback quality were concerning.

93.3% of participants felt written examination study groups led by higher trainees and consultants were relevant if available, 66.7% felt peer-led groups were relevant, and 66.7% felt happy with both online or in-person sessions. 100% of CASC participants strongly valued consultant examiner feedback.

Post evaluation, 100% favoured continuing CASC groups; and 87% favoured continuing written examination groups. 100% of CASC participants agreed or strongly agreed perceiving improvements in examination confidence, preparedness, and syllabus knowledge; and 75% of written examination participants strongly agreed perceiving improvements in these areas, satisfying project aims.

**Conclusion:**

Continuing peer-led group study initiatives was deemed worthwhile and successfully improved perceived examination confidence, preparedness, and syllabus knowledge. This represents a relatively inexpensive solution which, if scaled nationally, could beneficially supplement deaneries’ existing MRCPsych programmes.

